# Experiences of Nursing Teams Responding to Crisis Situations in the Psychosocial Care Network Services

**DOI:** 10.3390/nursrep16060198

**Published:** 2026-06-09

**Authors:** Marciana Fernandes Moll, Lucas Duarte Silva, Giovana Pires Borges, Ana Paula Rigon Francischetti Garcia, Erika Christiane Marocco Duran, Joaquim Manuel de Oliveira Lopes, Vanessa Pellegrino Toledo

**Affiliations:** 1 Nursing School of the State University of Campinas, Tessália Vieira de Camargo Street, 126, Zeferino Vaz University City, Campinas 13083-887, SP, Brazil; g257120@dac.unicamp.br (G.P.B.); apgarcia@unicamp.br (A.P.R.F.G.); ecduran@fenf.unicamp.br (E.C.M.D.); vtoledo@unicamp.br (V.P.T.); 2 Nursing School, Carajás College, 8 VP Avenue, Block 32, Section 16, Lot 02 (Special Section), Nova Marabá Neighborhood, Maraba 68508-150, PA, Brazil; lucas.duarte@carajasedu.com.br; 3 Nursing School of the University of Lisbon, Dom João II Avenue, Lot 4.69.01, 1990-096 Lisbon, Portugal; joaquimlopes@esel.pt

**Keywords:** psychiatric nursing, risk, nursing, mental health, perception

## Abstract

**Background/Objectives**: The escalation of psychiatric symptoms can pose risks to the safety of the patients and members of the health care team, particularly nursing staff who are in direct and constant contact with these patients whilst providing care. This study aimed to describe the reality experienced by nursing teams responding to crisis situations in Psychosocial Care Network services. **Methods**: Action research was conducted, for which data were collected using the focus group technique, with the participation of 10 to 11 nursing professionals. Twelve sessions were held using this technique, two for each of the six groups. For data analysis, full transcription and coding were performed using the Atlas TI Software (Version 23) to identify themes. Data analysis was developed using the thematic analysis technique. **Results**: The identified data categories are as follows: professionals’ perceptions of nursing team performance in caring for people in crisis; meanings attributed by professionals to situations of aggression during crises; and needs to be met for better management of people in crisis. **Conclusions**: Decentralizing responsibilities among the team and within the Network, including interdisciplinary care, makes it possible to provide comprehensive care for people in crisis. It is necessary to put integrated measures in place to safeguard the health of nursing staff working in Psychosocial Care Network services.

## 1. Introduction

In the field of health, a crisis is defined as a situation characterized by an inability to self-preserve and self-care, posing imminent danger to the individual or others and limiting their functionality in home and work environments [1]. Another definition describes a crisis as a specific individual moment in which behavioral manifestations affect, to varying degrees, the daily lives of the individual and those around them, with these behavioral manifestations requiring intervention by mental health services [2].

For the purpose of this study, the multidimensional concept of crisis will be addressed based on the “exacerbation of psychiatric symptoms” involving delusions, visual and auditory hallucinations, aggression, psychomotor agitation, disorganized thinking or behavior, and confused and incoherent speech, among others [3,4]. However, it is important to note that although this definition is fundamental for identification and immediate clinical intervention, it does not exhaust the subjective experience of crisis, which involves psychosocial and existential aspects included in other definitions.

Considering these conceptual bases, a crisis can be understood as a critical situation of rupture, acute imbalance, and instability that requires an urgent decision. In this context, nursing professionals have expressed that it is challenging to care for people with aggressive behavior, especially when performing interventions such as chemical, physical, or mechanical restraint, which are considered repressive and non-therapeutic practices, contradicting the principles of Psychiatric Reform [5,6,7,8,9].

Care practices aimed at people in crisis are predominantly provided by the nursing team, who face precarious and challenging situations, especially due to the lack of institutional resources and continuing education, leading to insecurity and insufficient support. These circumstances justify the reproduction of asylum practices, even though the nursing team has the potential to promote change [5,6,7,8,9]. In this sense, it should be noted that the number of hospitalizations reached 626,469, according to preliminary data from DataSUS filtered by the specialty “psychiatry” under the “urgent” care category from January 2020 to July 2025 [10,11]. This represents another challenge that seems to weaken the routine of health services, which are disrupted by the instability of people in crisis, and also the integrity of the nursing team, which is subject to experiencing situations of violence.

Given this scenario, it is important to recognize the perceptions of nursing professionals regarding the care of people in crisis in the Psychosocial Care Network (RAPS) services, as the RAPS is the central axis of coordination between different health services, whose objective is to ensure that users have access to accessible and comprehensive care at different levels of health care. In this context, nursing aims to provide care, management, and educational actions [12,13,14].

Although Brazil is a developing country, the state of mental health services is similar to that of Germany, as both prioritize social reintegration, valuing care in freedom through community-based services. However, in Germany, the right to care is consolidated, as there are resources favoring the development of safe practices for both users and professionals during crises, meaning efforts are focused on refining access and quality.

This same reality does not extend to India, which is also a developing country faced with obstacles to building a mental health system from scratch: a huge population, insufficient resources, limited funding, and a cultural battle against stigma. Its legislative advances, such as the Mental Health Act of 2017, which aims to protect patient rights, show a promising direction, but national-level implementation remains a major challenge.

As a result, India’s mental health system faces major limitations, such as a shortage of qualified professionals and low health insurance coverage, which hinders access and places financial burdens on families. Infrastructure is centralized in large cities, and the public system faces difficulties in integrating mental health into primary care. Furthermore, cultural stigma and negative views of mental disorders represent significant barriers that persist even after the National Mental Health Program and the Kiran Helpline [15,16,17,18,19,20,21], and this is also part of the Brazilian reality.

Considering Brazil has access to mental health care comparable to that of a developed country but experiences precarious conditions similar to those of a country that is still in the process of building its mental health network, it is necessary to highlight the reality of crisis management nursing teams in RAPS services.

This study aimed to describe the reality experienced by nursing teams in responding to crisis situations in RAPS services.

## 2. Materials and Methods

This study is part of a larger project, Action Research: Implementation of the Nursing Process in the Psychosocial Care Network, conducted in a municipality in the interior of São Paulo, Brazil.

This is an action research study, characterized as empirical social research, developed in close association with an action or the resolution of a collective problem, in which researchers and participants representative of the situation are involved in a cooperative or participatory manner [22]. Its use in nursing has expanded, especially in public health, hospitals, obstetrics, psychiatry, and teaching [23].

As this is a method with an original approach that aims to change routines, implement services, and modify the behavior of a group of individuals who become jointly responsible for the action taken, it is appropriate for the development of research in the field of nursing and in the present study [23].

This research represents a subset of this larger study and involved an exploratory study using a qualitative approach. Data collection was performed using a Focus Group (FG), a technique used in several studies, with good results in the field of nursing and health, since it facilitates discussions, the exchange of practical experiences, and the development of interventions [24,25].

The FG comprised 10 to 11 participants; a moderator (researcher) who was responsible for presenting the topics and deepening the discussion, in addition to mediating discussions that could cause a competitive environment; and an observer (researcher) who took notes during the session to later contribute to the data analysis [24]. The FG followed the following methodological steps: planning, conducting sessions, and data analysis [26].

Each FG participated in two sessions, during which they were presented with the following trigger questions. FG 1: Maria was recently hired at your workplace, and you need to introduce her to the nursing process that is developed there. What would you say to her? FG2: Sophia is a nurse who is trying to implement the nursing process in the mental health service where she works. Considering your knowledge on the subject, what suggestions would you give her about the necessary theory?

Twelve sessions were held using this technique: two for each of the six groups formed (the total number of participants was 64), in person and in a room reserved to ensure participant privacy. During these sessions, notes were taken by the rapporteur, and the participants’ statements were recorded. These were duly authorized before the start of each session.

For the analysis of the collected data, full transcription and coding were performed using the Atlas TI Software (version 23) to identify themes.

Data analysis was developed using the thematic analysis technique [27], which allows for a better understanding of the discourse, as it analyzes a social phenomenon. Thematic analysis favors the synthesis of nuclei that constitute communication and the construction of representations, thus allowing for better assimilation of the data in the analysis process [27].

Six steps were applied to understand the discursive context: (1) familiarization with the data (exhaustive reading of the discourses); (2) code generation (systematic coding of relevant and important data); (3) search for themes (grouping of selected codes for transformation into possible themes); (4) continuous review of themes and identification of the possibility of new themes being synthesized; (5) definition of themes, related to the analysis and refinement of the specificities of each theme; and (6) production of a final report (self-explanatory interpretation with aggregation of data and empirical categories) [27].

The thematic categories shown in Appendix A were elaborated based on the development of the first five steps.

The results obtained were discussed in light of the scientific literature. This research was approved by the Research Ethics Committee of the State University of Campinas, under opinion No. 6,971,386. All participants authorized the recording of the sessions and, to ensure anonymity, were identified by the letter E, for nursing, followed by an Arabic numeral.

## 3. Results

Based on the information provided by the participants, it was possible to classify the data into three categories: (1) professionals’ perceptions of the nursing team’s performance in caring for people in crisis; (2) meanings attributed by professionals to situations of aggression during crises; and (3) needs to be met for better management of people in crisis.

### 3.1. Professionals’ Perceptions of the Nursing Team’s Performance in Caring for People in Crisis

It was mentioned that, when caring for people in crisis, it is essential to recognize that the conditions are not only limited to psychiatric disorders, but also include clinical and neurological manifestations, such as convulsive and respiratory crises, adverse reactions to medications, and cardiorespiratory arrest.

Considering that these conditions represent crises, there was a discussion about the need to prepare the nursing team and other professionals to provide urgent and emergency care.

“When I started, there was a situation where a patient was choking, not only the nursing staff, but the occupational therapist who was there at the time, the psychologist […] one of us performed the Heimlich maneuver, we performed first aid there.”

(GF1, G1E1)

“There are patients who develop syndrome, they convulse […] patients who come from outside with injuries, traumatic brain injury…”

(GF1, G1E6)

These statements reinforce the need for a broader and more comprehensive view of the care process offered in RAPS services, requiring nurses to combine clinical and psychiatric knowledge and thus provide care based on the individual needs of the patient.

“Harm reduction stopped until the day the guy stopped in front of us […]. Everyone panicked, and I already worked with that, so at least I knew what to do. Because we are at CAPS, people think they don’t need to know.”

(GF2, G5E1)

In order to qualify nursing practice for the care of people in crisis, the importance of valuing the user’s history of previous illnesses was pointed out, so that it could be included in the care process, ensuring care based on safety and comprehensiveness.

“Most residents have underlying diseases, right? I thought about a complication. For respiratory assessment, a cardiac arrest.”

(GF2, G3E10)

“It’s not just scheduled medication, there are antibiotics that come from outside, with treatment…”

(GF1, G1E6)

The administration of medications requires significant attention from the nursing team, who must carefully monitor for possible drug interactions, adverse effects, and reactions to ensure therapeutic efficacy and patient safety. In this regard, some participants expressed that many users of RAPS services use multiple illicit and licit drugs that may predispose them to urgent or emergency situations.

“They could overdose there.”

(GF1, G2E3)

“In this case, if he accepts the medication, I even explain […] it’s an anxiolytic, it will sedate him, he will sleep. So I have to explain, right, the what this medication will do, his reaction.”

(GF2, G2E2)

Considering the severity of the condition of people in crisis, it may be necessary to activate other points of care in the Psychosocial Care Network (RAPS), and professionals mentioned that the Mobile Emergency Care Service (SAMU) is a support mechanism that has been strengthening networking and expanding the effectiveness of the care provided.

“[…] an complication may occur, the user may present a syndrome, or withdrawal symptoms, so we will provide nursing care and if it is a very serious case, we call SAMU.”

(GF1, G6E4)

“Our resident had a respiratory crisis at the beginning of the shift. His was saturating at 85. I told the caregiver who was with me that he was not well, and I called the emergency services. I accompanied him, but I returned because because of the other residents.”

(GF2, G3E7)

“The ambulance was called to provide all possible support to the patient who was there suffering from choking.”

(GF1, G1E1)

In addition to coordinating with emergency medical services, another tool considered effective by participants in some crisis situations was dialogue, which made it possible to reduce tensions, establish bonds of trust, and promote a safe therapeutic environment. To this end, listening and communication are used.

“With most users, I don’t need to restrain them, because just by talking to them talking to them […] when I arrived, I managed to contain this aggression in conversation, when I come back, he is already restrained because he was aggressive. It is not a pleasant environment for him to be in, inside the hospital.”

(GF2, G2E7)

Another point highlighted by the professionals was the importance of avoiding contact between stable patients and people with psychomotor agitation or other clinical crises, and even those who are being restrained.

“As we have some new arrivals, and as we are going to deal with those in crisis, with the others who are in a certain normal state.”

(GF1, G3E5)

“Usually when one person goes into crisis, the others seem to as well.”

(GF1, G3E3)

Given the above, it is clear that professionals realize that caring for people in crisis requires technical and emotional preparation on the part of the nursing team because, in addition to psychiatric manifestations, clinical and neurological conditions may occur that require clinical knowledge and coordination between health services. The statements highlight the importance of a comprehensive and interdisciplinary approach, as well as coordination with other RAPS devices. In addition, important elements for establishing a therapeutic relationship are valued, such as dialogue and skilled listening to reduce tensions and create a safe and humanized environment.

### 3.2. Meanings Attributed by Professionals to Situations of Aggression During Crises

For professionals, aggression resulting from an acute psychiatric crisis is a routine event that poses physical and psychological risks to the integrity of the nursing team.

“So, like it or not, one day it will be Maria’s turn to be assaulted, threatened […] She has to be aware of the risks she will face, but we try our best to welcome her […] take her out of the scene, take her out of the spotlight, but it will happen.”

(GF1, G3E3)

“Okay, but I might get hit. It’s normal for me to get hit, fine.”

(GF2, G6E3)

The unpredictability of crises also appeared in the statements of some participants, who pointed out that staff training does not exempt them from being surprised, and this causes them to remain constantly vigilant.

“He wasn’t showing any signs that he would do anything […] everyone was there that day there, and we couldn’t do anything to predict it, to prevent it from happen.”

(GF2, G6E3)

“Because we’re in a crisis environment, right? Suddenly everything is fine, and suddenly everything is not okay, right?”

(GF2, G6E7)

The patient’s aggression can manifest itself indiscriminately, affecting any member of the health care team present at the site, as explained in the following statement:

“It will happen countless times. It will happen on the weekend, it will happen at night, it will happen at 8 in the morning, it happens with the cleaning staff, the girls at the reception desk, it is not selective.”

(GF1, G3E3)

However, for professionals, situations of aggression are not intentional and correspond to a physical manifestation of intense psychological suffering.

“The three times I was assaulted, I am not angry, because they are patients who were hitting people, they were in crisis, it wasn’t personal. So I have no reason to be angry, to fight back, right? You’re at CAPS.”

(GF1, G4E1)

“Then you stop, you think, you see and you even understand that person, the crisis they were going through at that moment, you know?”

(GF1, G3E3)

In general, professionals understand aggression during psychiatric crises as an inherent and routine phenomenon in mental health care settings, and recognize that the unpredictability of crises keeps them in a constant state of vigilance. Furthermore, it was evident that participants consider episodes of physical aggression to be the result of intense suffering for the patient rather than deliberate actions, which necessitates emotional preparation, sensitivity, and ethics on the part of professionals when dealing with crises.

### 3.3. Needs to Be Met for Better Management of People in Crisis

It was evident among professionals that the proper management of a person in psychiatric crisis requires that everyone on the health team have the knowledge and skills to intervene appropriately.

“It’s just that the crisis, within CAPS, belongs to everyone, to everyone.”

(GF1, G4E1)

“Come on, it’s a crisis, right? As far as I know, a crisis belongs to everyone.”

(GF1, G4E5)

In this context, it is mentioned that cohesion among professionals is a determining factor for successful management, which requires integrated action, mutual trust, and clear communication.

“I think the team has to be very cohesive in a crisis, right? Because responding to a crisis, where one prescribes, the other says no… […] it doesn’t work. This work is impossible with these people, this way.”

(GF1, G4E5)

“There have been some really bad crises there, where the doctor said ‘give haldol with promethazine,’ and the psychologists wouldn’t let me administer the medication, which was already drawn up in my hand.”

(GF2, G6E3)

This report draws attention to the need for the multidisciplinary team to agree on a protocol that integrates all its members, since the management of a psychiatric crisis should not be seen as the exclusive responsibility of a single professional or category, but rather as a collective responsibility of the entire team. In this sense, the nursing team reports feeling overwhelmed, as the very nature of the profession means that these professionals take control and act on the front line when caring for people in crisis.

“But what we see is that in times of crisis, everyone hides in a corner and the nurses are left in the middle.”

(GF1, G4E1)

“If you do a survey within mental health, of the accidents, it’s only nursing that gets beaten up. Why don’t the rest get hurt?”

(GF1, G4E5)

Given the intense involvement of nursing professionals in caring for people in crisis, it is common for them to be assaulted, which leads to a negative and impactful experience. However, there is no institutional support for them, which means they have to find their own resources to deal with and overcome these experiences.

“So, I think Maria has to be aware of this if she is going to be able to deal with this type of aggression. It’s something she’ll have to learn to deal with.”

(GF1, G3E3)

“I thought, what am I going to say to all the patients in crisis? Do I have to be angry? No. They didn’t even know what they were doing.”

(GF1, G4E1)

For some participants, it is important that professionals who are victims of violence while caring for people in crisis are welcomed.

“The problem is not suffering violence, but what is done to us afterwards that? Which is absolutely nothing, okay, nothing.”

(GF1, G3E3)

Another important point refers to communication among team members for sharing information about people who are being assisted and are at greater risk of developing aggressive behavior, as well as disclosing the factors that predispose them to crises (emotional and social occurrences, among others) and the signs of instability that precede crises.

“I would like to be warned, for example, if there is an agitated, aggressive, a patient who has already experienced something more serious within CAPS or outside CAPS.”

(GF1, G3E2)

The need for training that addresses the care of people in crisis was also added.

“I also feel a need for us to increasingly prepare ourselves to deal with crises, right? So that we don’t also end up in crisis along with the patient, because otherwise everyone ends up in crisis there, managing a crisis, right?”

(GF1, G4E2)

Therefore, the participants emphasized that the proper management of people in crisis requires strategic alignment of the health care team, in which everyone has the knowledge, skills, and coordination to act safely and decisively, knowing how and where to act and reducing the burden often assumed by the nursing profession. Through consistency, connection, and clear communication among the team, it is possible to promote shared responsibility and strengthen technical preparedness. Furthermore, the statements point to the lack of institutional support during and after situations of aggression, highlighting the need for emotional support for professionals, as well as investments in training and continuing education activities.

## 4. Discussion

In the present study, it was evident that people with mental disorders may present clinical comorbidities that destabilize them and may represent a crisis. These people are prone to presenting various associated medical conditions, such as hypertension, diabetes, and infectious diseases [28]; therefore, it is essential to carefully assess and offer, in addition to appropriate psychiatric treatment, the necessary clinical care. Although there is no direct relationship between NCDs and mental disorders, it is possible to identify the signs and symptoms of these diseases through risk factors such as poor diet and physical inactivity [29].

It should also be noted that during hospitalization, people with schizophrenia are at an additional risk of developing respiratory disease due to staying indoors and poor oral hygiene [30]. Furthermore, it was noted that people with psychotic disorders, regardless of medication use, were predisposed to overweight and obesity and high blood pressure levels without having a diagnosis of systemic arterial hypertension [31].

In the present study, the frequent presence of SAMU was noted to contribute to the care of people in crisis. This device plays an essential role in redirecting care to people with mental disorders, often being the gateway for these people in crisis to health services that have better technological and professional density for stabilizing their condition [32].

However, continuous awareness-raising is necessary, since urgent and emergency care in psychiatric cases is mechanistic and does not consider the subjective needs of the person, which highlights the need for professional training so that teams have a cohesive understanding of the flows and functioning of RAPS, as well as for the development of appropriate protocols for meeting psychiatric demands [33].

It should be emphasized that nurses’ contact with psychiatric emergency care occurs sporadically during their undergraduate studies [33], which is consistent with statements of insecurity and unpreparedness by some professionals in this investigation.

There is also concern among nursing professionals about keeping people stable in a location other than where the crisis is occurring, and this was pointed out in a recent study [34] as necessary to not cause fear or expose people to aggression.

Furthermore, dialogue was mentioned as a tool for crisis management, and this behavior corroborates with research [35], which mentions assertiveness as a behavior that can alleviate the suffering of the person in crisis and contribute to the creation of bonds and trust. Furthermore, it is stated that empathy and non-judgmental attitudes contribute to stabilization and reduce the need for practices such as restraint [35].

In general, the results of this investigation show that crises are a daily occurrence in mental health services, carrying a risk of physical aggression, which corroborates the assertion that aggression against health professionals is a common occurrence, especially in mental health services, and this needs to be recognized by professionals and managers [36].

In this context, events that trigger aggression against health professionals working in psychiatric services should be analyzed in order to establish strategies for combating and preventing it, since violence can cause physical and psychological harm to workers [37]. This reality was highlighted in this investigation and was also addressed in scientific research that shows that professionals, especially nurses, fear for their physical safety and are even uncomfortable when caring for people in crisis [38].

The unpredictable behavior of people in crisis was also mentioned by the participants, and scientific evidence points out that nursing professionals, when working in mental health services, need to understand that it is often impossible to predict the behavior of the people being cared for, with the risk of violence being intrinsic to providing care [37].

Given this, training alone is not effective, as the participants noted. It is necessary for nursing professionals to develop maturity and emotional control, and if they do not feel capable, they should seek help from other professionals [39]. Thus, this study emphasizes the need to remain vigilant while providing nursing care in mental health services. One of the strategies to ensure this is through the promotion of a safe and risk-free environment for workers, as well as the implementation of risk management practices [37].

The professionals participating in this study recognized that aggression on the part of patients is not intentional but reflects their psychological suffering, and this is reinforced by scientific findings that point to a relationship between impulsivity and aggressiveness in people diagnosed with mood disorders [40]. Furthermore, all violence is a tragic externalization of an unmet need [41].

Given this context, nursing professionals do not feel prepared to perform actions that require such relational skills in the management of psychiatric crises, and this disqualifies the care provided [42]. The participants in this study mentioned that the lack of cohesion among professionals and the presence of only nursing professionals in crisis control procedures also represent determinants for the quality of management.

The need to decentralize the nursing team’s role in crisis care and focus on the team’s bond with the subject is essential [43] because, with the integration of diverse knowledge, we can establish safe and ethical conduct in crisis management. For this to occur, the need to establish protocols as instruments that systematize care was mentioned, assisting in management, reducing unnecessary variations in practices, and supporting health professionals in executing procedures [44].

Importantly, this investigation revealed the absence of a protocol and that interventions are carried out exclusively by the nursing team, exposing them to overload, emotional distress, and physical violence. In this sense, recent studies [45,46] clarify that the lack of policies focused on the mental health care of the professionals themselves and the lack of institutional recognition generate a cycle of exhaustion and emotional distress that weakens cohesion among the team.

Physical violence against professionals working in psychiatry is frequent and causes physical and psychological damage that has historically been naturalized and rationalized. This normalization is responsible for increasing fear, stress, and irritability among these professionals. To reduce these conditions, it is recommended to provide psychological assistance after incidents of aggression so that feelings such as sadness, injustice, helplessness, fear, and guilt resulting from the experiences of violence suffered can be addressed [47].

Among the strategies identified for managing people in crisis are professional training, sharing information about people who are being assisted and at greater risk of developing aggressive behavior, and disseminating information about the factors that predispose people to crises and the signs of instability that precede crises. Individual and team training is periodically identified as favorable for the early identification of signs indicative of crises and the establishment of interdisciplinary therapeutic approaches that ensure greater safety and less overload [39].

## 5. Conclusions

The experiences of the nursing team in managing people in crisis in the Psychosocial Care Network services comprise a complex, multidimensional process, heavily influenced by structural, organizational, and subjective challenges.

The findings showed that nursing staff play a central role in caring for people in crisis, working predominantly on the front line, which repeatedly exposes them to situations of overload, insecurity, and physical and emotional violence. Although professionals recognize aggression as an expression of intense psychological distress and not as an intentional attitude on the part of the user, the normalization of these experiences, associated with the absence of institutional support and post-aggression care strategies, weakens the mental health of workers and compromises the quality of care provided.

The fact that the investigation took place in only one city in the interior of São Paulo represents a limitation of this investigation, especially since Brazil is a country with extensive territorial density. However, our results highlight the urgency of institutional investments that promote the decentralization of responsibilities among the team and within the Network itself, the strengthening of interdisciplinary action, and care for the physical and mental health of professionals. It is suggested that future studies investigate and emphasize the importance of promoting integrated, ethical actions based on the principles of care in freedom, ensuring protection, dignity, and comprehensiveness for both users and health workers.

## Figures and Tables

**Figure A1:**
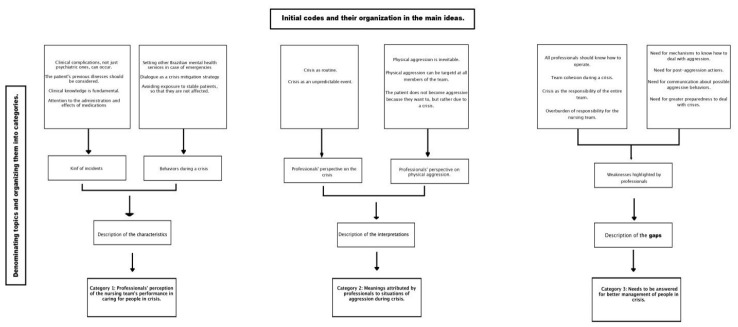
Experiences of Nursing Teams Responding to Crisis Situations. Jundiaí/SP, 2025.

## Data Availability

The data presented in this study are available on request from the corresponding author. The data are not publicly available due to privacy or ethical restrictions.

## Public Involvement Statement

Participants voluntarily took part in focus groups, and this research was approved by the Ethics Committee (Opinion No. 6,971,386).

## Guidelines and Standards Statement

This manuscript was drafted in accordance with the COREQ (Consolidated Criteria for Reporting Qualitative Research) for qualitative research.

## Abbreviations

The following abbreviations are used in this manuscript:

FG	Focus Group
RAPS	Psychosocial Care Network
SAMU	Mobile Emergency Care Service
